# A rare presentation of systemic lupus erythematosus: diffuse alveolar hemorrhage in an infant

**DOI:** 10.1186/1546-0096-12-S1-P315

**Published:** 2014-09-17

**Authors:** Kubra Ozturk, Demir Kursat Yıldız, Zeynep Seda Uyan, Zelal Ekinci

**Affiliations:** 1 Department of Pediatrics Rheumatology, Kocaeli Univertsity Faculty of Medicine, Kocaeli, Turkey; 2 Department of Medical Pathology, Kocaeli Univertsity Faculty of Medicine, Kocaeli, Turkey; 3 Department of Pediatric Pulmonology, Kocaeli Univertsity Faculty of Medicine, Kocaeli, Turkey

## Introduction

Systemic lupus erythematosus (SLE) is a multisystem autoimmune disease characterized by a variable clinical picture and serological abnormalities. Children and adolescents generally have a more severe disease presentation; develop disease damage more quickly than adults with SLE. Diffuse alveolar hemorrhage (DAH) is a rare life-threatening complication in SLE. In this report a case of infantile onset SLE with DAH and negative ANA is presented.

## Objectives

In this report a case of infantile onset SLE with DAH and negative ANA is presented.

## Methods

### Case report

A 1-year-old boy presented with petechia on his legs. Physical examination was notable for ecchymoses and petechiae in the lower extremities. He had a 2/6 systolic murmur and hepatomegaly. Arterial blood pressure was 121/81 mm/Hg (> 95 th percantile). On admission, the patient’s hemoglobin was 7.2 g/dL, white blood cells (WBC) 15900/mm^3^, platelets 50200/mm^3^, BUN 25 mg/dL, creatinine 0.53 mg/dL, alanine aminotransferase 120 U/l, aspartate aminotransferase 99 U/l. Urinalysis revealed Ph 5.0, density 1020, blood 3+ and protein 2+. Spot urine protein creatinin ratio was 5.7 mg/mg. Complements C3 was 23 mg/dL (90-180 mg/dL) and C4 was 2.9 mg/dL (10-40 mg/dL). Direct Coombs was +4 positive. Bone marrow aspiration revealed hypercellular bone marrow with an increased myeloid series. Echocardiography revealed pericardial effusion.With the preliminary diagnosis of SLE, a renal biopsy was performed. Diffuse global proliferative nephritis (Class IV-G(A)) was observed. Glomerular deposits of IgG, IgM and complement C3 was seen with immunofluorescence microscopy. However, antinuclear antibody (ANA) and anti-double-stranded DNA were negative.

## Results

The patient was diagnosed as SLE with Systemic Lupus International Collaborating Clinics (SLICC) criteria which were reported to be more sensitive than ACR (American College of Rheumatology) criteria. The initial SLE Disease Activity Index (SLEDAI) was 15. Intravenous methylprednisolone was administered at a dose of 30 mg/kg/day. In the second day of treatment he suddenly developed dyspnea. Chest radiograph demonstrated diffuse alveolar shadows in both lungs without cardiyomegaly. He was transferred to the intensive care unit with the diagnosis of acute respiratory distress syndrome (ARDS) and supported with mechanical ventilation. During this support severe lung bleeding was inspected from the endotracheal tube. Intravenous cyclophosphamide 500 mg/m^2^ was added to therapy and response to treatment was observed in a few days. He was successfully weaned off ventilator support after 7 days. In his 4th follow-up month, he is in remission with monthly cyclophosphamide and prednisolone.

## Conclusion

Children and adolescents generally have a more severe disease presentation; develop disease damage more quickly than adults with SLE. Diffuse alveolar hemorrhage (DAH) is a rare life-threatening complication of SLE. It is an uncommon complication with estimates ranging from < 2 to 5.4 % in cohorts of lupus patients. DAH usually occurs in patients with established diagnosis of SLE; however, cases have been reported where DAH was initial presentation of SLE. DAH can often mimic severe pneumonia or ARDS. Optimal management of DAH has not been established. Use of cyclophosphamide has been linked to better survival as in our case.This case is having a combination of the rarely known presenting features of SLE as infantile onset, DAH and negative ANA.

## Disclosure of interest

None declared

